# Quorum Sensing Inhibiting Activity of Cefoperazone and Its Metallic Derivatives on *Pseudomonas aeruginosa*

**DOI:** 10.3389/fcimb.2021.716789

**Published:** 2021-09-30

**Authors:** Nourhan G. Naga, Dalia E. El-Badan, Heba S. Rateb, Khaled M. Ghanem, Mona I. Shaaban

**Affiliations:** ^1^ Department of Botany and Microbiology, Faculty of Science, Alexandria University, Alexandria, Egypt; ^2^ Department of Pharmaceutical and Medicinal Chemistry, Pharmacy College, Misr University for Science and Technology, Cairo, Egypt; ^3^ Department of Microbiology and Immunology, Faculty of Pharmacy, Mansoura University, Mansoura, Egypt

**Keywords:** quorum sensing inhibition, cefoperazone, *Pseudomonas aeruginosa*, virulence factors, LasR, cefoperazone derivatives, cefoperazone cobalt complex, cefoperazone-iron complex

## Abstract

The last decade has witnessed a massive increase in the rate of mortalities caused by multidrug-resistant *Pseudomonas aeruginosa*. Therefore, developing new strategies to control virulence factors and pathogenicity has received much attention. One of these strategies is quorum sensing inhibition (QSI) which was developed to control *Pseudomonas* infection. This study aims to validate the effect of one of the most used β-lactam antibiotics; cefoperazone (CFP) and its metallic-derivatives on quorum sensing (QS) and virulence factors of *P. aeruginosa.* Assessment of quorum sensing inhibitory activity of CFP, cefoperazone Iron complex (CFPF) and cefoperazone Cobalt complex (CFPC) was performed by using reporter strain *Chromobacterium violaceum* ATCC 12472. Minimal inhibitory concentration (MIC) was carried out by the microbroth dilution method. The influence of sub-MICs (1/4 and 1/2 MICs) of CFP, CFPF and CFPC on virulence factors of *P. aeruginosa* was evaluated. Data was confirmed on the molecular level by RT-PCR. Also, molecular docking analysis was conducted to figure out the possible mechanisms of QSI. CFP, CFPF, and CFPC inhibited violacein pigment production of *C. violaceum* ATCC 12472. Sub-MICs of CFP (128- 256 μg/mL), and significantly low concentrations of CFPC (0.5- 16 μg/mL) and CFPF (0.5- 64 μg/mL) reduced the production of QS related virulence factors such as pyocyanin, protease, hemolysin and eliminated biofilm assembly by *P. aeruginosa* standard strains PAO1 and PA14, and *P. aeruginosa* clinical isolates Ps1, Ps2, and Ps3, without affecting bacterial viability. In addition, CFP, CFPF, and CFPC significantly reduced the expression of *lasI* and *rhlI* genes. The molecular docking analysis elucidated that the QS inhibitory effect was possibly caused by the interaction with QS receptors. Both CFPF and CFPC interacted strongly with LasI, LasR and PqsR receptors with a much high ICM scores compared to CFP that could be the cause of elimination of natural ligand binding. Therefore, CFPC and CFPF are potent inhibitors of quorum sensing signaling and virulence factors of *P. aeruginosa*.

## 1 Introduction

*Pseudomonas aeruginosa* is an encapsulated, Gram-negative, rod-shaped opportunistic pathogen that infects plants, animals, and human (Borges et al., 2018). *P. aeruginosa* can colonize critical body organs such as lungs, urinary tract, and kidney with fatal pathological effects especially on immunocompromised patients (Turkina and Vikström, 2019).

Treatment of *P. aeruginosa* infections by antibiotics is getting ineffective due to the worldwide spread of multi-drug resistant isolates. In addition, *P. aeruginosa* possesses diverse virulence factors including biofilm, pyocyanin, elastase, aminopeptidase, chitinase, protease, lipase, alginate, and hydrogen cyanide (Schuster and Greenberg, 2006). All these virulence triats assist the spreading and dissemination of *Pseudomonas* infection in different organs.

*Pseudomonas* virulence behaviors are synchronized by QS signaling system. Four fundamental QS pathways have been distinguished in *P. aeruginosa*. First, *lasI/lasR* system which uses 3-oxododecanoyl-L-homoserine lactone (3-oxo-C12-HSL) as an autoinducer. Second, *rhlI/rhlR* system which utilizes N-butanoyl homoserine lactone (C4-HSL) as an autoinducer (Pesci et al., 1997). Both systems are linked to the third system, *Pseudomonas* quinolone signal (PqsABCDE/PqsR), which responses to 2-heptyl-3-hydroxy-4-quinolone autoinducer (Déziel et al., 2005; Dubern and Diggle, 2008). Fourth, the integrated quorum sensing, (AmbBCDE/IqsR), which uses the autoinducer 2-(2-hydroxyphenyl) thiazole-4-carbaldehyde (Lee & Zhang, 2015; Rampioni et al., 2016). These four QS signaling systems are interconnected to regulate the expression of various virulence genes in *P. aeruginosa* (Parsek and Greenberg, 2000).

Inhibition of quorum sensing is one of the most promising approaches to counteract *Pseudomonas* virulence factors and associated pathogenic infection. QSI with different modes of action has been developed (Shaaban et al., 2019; Proctor et al., 2020). There are different natural and synthetic compounds that have been identified as QSIs. Natural compounds such as ascorbic acid (Vitamin C) (El-Mowafy et al., 2014), zingerone (Kim et al., 2015), curcumin (Tyagi et al., 2015), alginates (He et al., 2014), and 1*H*-pyrrole-2-carboxylic acid (Hassan et al., 2016) have been identified as QSIs (Ahmed et al., 2019). Moreover, synthetic QSIs have been reported including halogenated furanones (Hentzer et al., 2002), benzothiazole (Gabr et al., 2015), isatin-β-thiocarbohydrazones (Gabr et al., 2018), and phenylalanine arginyl β-naphthylamide (El-Shaer et al., 2016).

In addition, various antimicrobial agents elicit anti-QS effects and eliminate QS-associated virulence factors. QSI activity of azithromycin (Tateda et al., 2001), doxycycline (Husain and Ahmad, 2013), and ciprofloxacin (Skindersoe et al., 2008) have been detected (Nalca et al., 2006). Moreover, β-lactams, ceftazidime, cefepime and imipenem have demonstrate QSI activity at sub-inhibitory concentrations (Skindersoe et al., 2008; El-Mowafy et al., 2017; Aleanizy et al., 2021).

CFP is a bactericidal third-generation cephalosporin possessing extended-spectrum activity against Gram-negative bacilli. Resistance to CFP has been developed where CFP was ineffective in the treatment of *Pseudomonas* infection. Thus, it is necessary to develop strategies to combat virulence factors without acquiring resistance. Masoud and coauthors have developed CFP metallic derivatives with antimicrobial activity against Gram-negative bacilli (Masoud et al., 2017). To our knowledge, QSI activity of CFP and its derivatives have not been evaluated. So, the aim of this study is to investigate the effect of CFP and its metallic complexes on QS circuits and virulence factors as a new strategy for combating *Pseudomonas* infection.

## 2 Materials and Methods

### 2.1 Bacterial Strains, Media, and Conditions

*Chromobacterium violaceum* ATCC 12472 reporter strain was used in the assay of QSI activity of CFP and its metallic complexes (McClean et al., 1997). The reporter strain was grown on Luria–Bertani (LB) media containing (tryptone, 10 g/L; yeast extract, 5 g/L; and NaCl, 10 g/L) at pH 7, and incubated at 28°C for 48 hours (Bertani, 2004). *P. aeruginosa* clinical isolates were isolated from urine samples and named Ps1, Ps2, and Ps3 according to the Helsinki declaration in handling, use and care of human subjects for medical research. Ethical approval was obtained from the Institutional Review Boards of Faculty of Medicine, Alexandria University, Egypt.

These clinical isolates were identified as *P. aeruginosa* according to laboratory biochemical standards. *P. aeruginosa* PAO1 and *P. aeruginosa* PA14 standard strains were also used for assessment of QSI effects of the tested chemical compounds as positive controls and *P. aeruginosa* PAO-JP2 (△*lasI*::Tn10, Tcr; △*rhlI*::Tn501-2, Hgr) is a QS double-mutant strain and has been used as a negative control (Pearson et al., 1997). All *P. aeruginosa* strains were cultivated in LB media and incubated overnight at 37°C.

### 2.2 Cefoperazone and Metallic Derivatives

CFP complexes with FeCl_3_.6H_2_O, CoCl_2_, MnCl_2_, NiCl_2_, and CrCl_3_ (Sigma Aldrich, USA) **(** **Table 1**) were prepared according to the method reported by Masoud and coauthors (Masoud et al., 2017). Each Metal chlorides (1 M) was dissolved in 40 mL ethanol and CFP (1 M) was dissolved in 40 mL double-distilled water. Then, metal chloride ethanolic solutions and CFP solutions were mixed in a molar ratio of 1:1. Each reaction mixture was refluxed for 4 hours and left overnight. The formed precipitate was filtered and washed with 10 ml ethanol 95% w/v, and dried in a vacuum desiccator over anhydrous CaCl_2_. The formed CFP metal complexes were confirmed by UV and thermal analysis (Masoud et al., 2017). Stock solutions of cefoperazone-metallic derivatives were prepared at a concentration 5 mg/ml in DMSO (20% v/v).

**Table 1:** Inhibition of violet pigment of *Chromobacterium violaceum* ATCC 12472 by cefoperazone and its metallic derivatives.

	Derivative	Code	Anti-QS zone (mm)
Cefoperazone	–	CFP	25
	FeCl_3_.6H_2_O	CFPF	33
	CoCl_2_	CFPC	34
	MnCl_2_	CFPM	–
	NiCl_2_	CFPN	–
	CrCl_3_	CFPR	–

### 2.3 QSI Assay of Cefoperazone and Metallic Complexes Using *C. violaceum* ATCC 12472

*C. violaceum* ATCC 12472 reporter strain was used to determine the QSI activity of CFP and different complexes. The culture of *C. violaceum* was propagated at 28°C with 200 rpm agitation in a shaking incubator for 48 hours. The double-layer culture plate method was performed where 15 mL of LB medium (2% w/v agar) was poured into the plates (9 cm). Solidified LB plates were overlaid with 8 mL of soft LB medium (1% w/v agar) containing 100 μL of *C. violaceum*, and the plates were left to completely solidify. Wells were cut in the agar using a sterile cork borer (10 mm diameter). CFP and its derivative CFPC, and CFPF (100 μL) at 1/2 and 1/4 MICs were loaded in the corresponding wells and the plates were incubated at 28°C. The inhibition zone diameter of the violet color of violacein pigment around each well was measured after 24 hours (McClean et al., 1997; McLean et al., 2004). In each plate, DMSO was applied in one well as a control.

### 2.4 Determination of Minimal Inhibitory Concentrations

Compounds that showed inhibition of violacein pigment production were selected. The minimal inhibitory concentrations of the selected compounds; CFP, CFPC, and CFPF were determined using the microtitre plate assay method. Muller Hinton broth (100 µL) was distributed in each well, 100 µL of the tested compound was added to the first well. Two-fold serial dilution of the tested compound was performed in the subsequent wells to have serial dilutions of 512, 256, 128, 64, 32, 16, 8, 4, 1, and 0.5 µg/mL. Each well was inoculated with 1 X10^5^ CFU of *P. aeruginosa* cultures Ps1, Ps2, Ps3, PAO1, PA14, and PAO-JP2. Wells containing media only and wells receiving media inoculated with the test strains were included in each plate as negative and positive controls, respectively. All plates were incubated overnight at 37°C and the microbial growth in each well was visually detected. The MIC was calculated as the lowest concentration that eliminated microbial growth compared to the positive control (CLSI, 2014). Sub-MICs of CFP, CFPC and CFPF were calculated, and the tested strains were propagated in the presence of sub-MICs of the tested compounds.

### 2.5 The Effect of Sub-Inhibitory Concentrations on Bacterial Growth

*P. aeruginosa* strains Ps1, Ps2, Ps3, PAO1, PA14 and PAO-JP2 were propagated in the presence of 1/2 MICs of CFP, CFPC and CFPF. Control untreated strains were also cultivated under the same conditions. In brief, LB broth media (25 mL) supplemented with 1/2 MIC of the tested compounds CFP, CFPC and CFPF was inoculated with overnight culture of Ps1, Ps2, Ps3, PAO1, PA14 and PAO-JP2 to achieve 0.05 OD 600 nm at zero time. From each mixture, 1 mL was collected at different time intervals and the OD 600 nm was estimated. In addition, viable counts of treated and untreated *P. aeruginosa* strains Ps1, Ps2, Ps3, PAO1, PA14 and PAO-JP2 were estimated after 18 hours using the pour plate method. The collected samples were diluted 1:10 and 1 mL of each dilution was inoculated in LB agar (15 mL) and distributed in 9 cm plate. The plates were solidified and incubated at 37°C for 18 hours. The count of bacterial cells of each sample was calculated as the viable *P. aeruginosa* colonies X dilution factor and represented as CFU/mL (Standards Australia, 1995).

### 2.6 Effect of Sub-MICs of the Tested Compounds on *Pseudomonas* Virulence Factors

CFP, CFPC and CFPF at 1/2 and 1/4 MICs did not exhibit any significant effect on microbial growth. So, they were assessed for their effect on different virulence factors of *P. aeruginosa*. The tested *P. aeruginosa* strains Ps1, Ps2, Ps3, PAO1, PA14, and PAO-JP2 (negative control) were propagated in the presence of 1/2 and 1/4 MICs of CFP, CFPC, and CFPF (Musthafa et al., 2012). The untreated strains were also grown under the same conditions. Assay of different virulence factors was performed in the presence and absence of the tested compounds (El-Mowafy et al., 2017).

#### 2.6.1 Pyocyanin Assay

Pyocyanin quantification was performed by using King’s A broth media. Overnight culture of *P. aeruginosa* strains was inoculated into 5 mL of King’s A media. Both untreated and treated cultures with sub-MICs (1/2 and 1/4 MICs) of CFP, CFPC, and CFPF were incubated at 37°C for 48 hours with shaking. Pyocyanin was extracted from the supernatant with 3 mL chloroform. The mixture was centrifuged at 3000 rpm for 10 min. Chloroform fractions were transferred to a new tube, then 1 mL of 0.2 M HCL was added, and the mixture was re-centrifuged for 5 min at 3000 rpm. The absorbance of the aqueous layer was measured at OD 520 nm. Pyocyanin concentration was calculated from the equation: pyocyanin concentration (µg/mL)= OD 520 nm × 17.072 (He et al., 2014; Saurav et al., 2016).

#### 2.6.2 Total Protease Production

The tested *P. aeruginosa* strains were inoculated in LB broth media supplemented with 1/2 and 1/4 MICs of CFP, CFPC, and CFPF and grown overnight at 37°C. The untreated strains were also grown under the same conditions. Cells were centrifuged and proteolytic activity of the treated and untreated *P. aeruginosa* isolates was measured (Rossignol et al., 2008). Proteolytic activity was determined using skimmed milk assay technique (Skindersoe et al., 2008; El-Mowafy et al., 2017). The assay relies on determining the change in the turbidity of skimmed milk accompanying protease activity. Skimmed milk was freshly prepared by dissolving 1.25 gm of skimmed milk in 100 mL sterile distilled water at 60°C. Then, 0.5 mL of culture supernatant was added to 1 mL of the prepared skimmed milk, and the mixture was incubated at 37°C for 1 hour. The degree of clearance of skimmed milk was determined by measuring the turbidity at OD 600 nm. The decrease in the OD 600 nm was indicated by the clearance of the skimmed milk with elevated proteolytic activity (El-Mowafy et al., 2014).

#### 2.6.3 Determination of Hemolysin Activity

The tested *P. aeruginosa* strains were propagated in the presence of 1/2 and 1/4 MICs of CFP, CFPC, and CFPF overnight at 37°C (Musthafa et al., 2012). The untreated strains were also grown under the same conditions. Cells were centrifuged and the hemolytic activity of treated and untreated *P. aeruginosa* isolates was measured (Rossignol et al., 2008). RBCs (obtained from sheep) were washed three times with sterile physiological saline and re-suspended in tris-buffer (pH 7.4, 0.025 M) to a final concentration of 2% v/v. For hemolysin assay, 700 μL of erythrocytes suspension was mixed with 500 μL of treated and untreated cell-free supernatants and incubated for 2 hours at 37°C. The suspension was centrifuged at 3000 rpm for 10 min at 4°C, and cell lysis was assessed by determining absorbance at OD 540 nm.

#### 2.6.4 Quantification of Biofilm Formation Using Microtiter Plate Assay

Flat bottomed polystyrene microtiter plates were used to evaluate slime production and biofilm formation. Overnight cultures of the tested *P. aeruginosa* strains were diluted with sterile LB broth to 0.5 McFarland. Treated and untreated cultures (100 μL) were transferred to each well and the plates were incubated for 24 hours at 37°C for mature biofilm formation. The content of each well was aspirated using Pasteur pipette and each well was rinsed three times with 200 μl of physiological saline. The plates were shaken to remove all non-adherent cells and the remaining attached bacteria were fixed with 150 μL of absolute methanol for 15 min, then, the plates were emptied and left to dry. Sessile cells bound to the wells were stained with 150 μL of crystal violet (1% v/v) for 10 min. Excess stain was rinsed off, and the plates were washed with water. The plates were air-dried and the dye bound to the wells was eluted with 150 μL of 33% (v/v) glacial acetic acid (Adonizio et al., 2008). The absorbance was measured at OD 490 nm using a microtiter plate reader (Diaket, ELISA plate reader).

### 2.7 Expression of QS Genes

The effect of CFP, CFPC, and CFPF at sub-MICs on the expression of QS genes *lasI* and *rhlI* in *P. aeruginosa* PAO1 was assessed by RT-PCR. The untreated and treated PAO1 with 1/2 MIC of CFP, CFPC, and CFPF were propagated, and cells were collected at the middle of exponential phase. Total RNA was extracted by Triazole reagent (Sigma Chemicals, USA). Chloroform (100 µL) was added to each sample, the tubes were incubated for 2–3 min at room temperature and centrifuged at 12.000 rpm for 15 min at 4°C. The aqueous phase was collected in RNase free Eppendorf tubes and chloroform step was repeated for complete purification of RNA. Isopropanol (300 µL) was added to each tube, the tubes were mixed for 1 min and centrifuged at 12.000 rpm for 15 min at 4°C. The supernatant from the previous step was removed leaving RNA pellet. The pellet was washed twice with 1 ml of ethanol 75% w/v. The sample was gently mixed, and centrifuged at 10.000 rpm for 5 min at 4°C. The supernatant was discarded, and RNA pellet was air dried for 10-15 min. The concentration and the purity for each RNA sample were determined using NanoDrop (ND-1000 Spectrophotometer, NanoDrop Technologies, Wilmington, Delaware, USA).

Complementary DNA (cDNA) was synthesized using Quanti-Tect Reverse Transcription kit (Qiagen, Germany) according to the manufacturer’s instructions. RT-PCR reaction mixture was composed of cDNA, 4 µL of the 5X FIREPol Eva Green, qPCR Mix (Solis Bio- Dyne, Estonia), 2 nM of each primer (**Table 2**), and RNAase free water to final volume 20 µL. RT-PCR was performed as follows: one cycle at 95°C for 15 min, followed by 40 cycles each cycle programmed as denaturation at 95°C for 15 s, annealing for 30 s and extension at 72°C for 30 s. RT-PCR was performed using a Rotor- Gene Q thermocycler (Qiagen, Germany). A negative control containing RNAase/DNAase-free water instead of cDNA was included in each run.

**Table 2:** Specific amplification primer sets for *P. aeruginosa* isolates.

Gene type	Gene name	Type of primer	Primer Sequence	Melting temp.	Amplicon size (bp)
**Reference gene.**	*rpoD*	Fw	5`**–**CGAACTGCTTGCCGACTT**–**3`	56°C	131
		Rev	5`**–**GCGAGAGCCTCAAGGATAC**–**3`		
**QS genes.**	*lasI*	Fw	5`**–**CGCACATCTGGGAACTCA**–**3`	56°C	176
		Rev	5`**–**CGGCACGGATCATCATCT**–**3`		
	*rhlI*	Fw	5`**–**GTAGCGGGTTTGCGGATG**–**3`	58°C	101
		Rev	5`**–**CGGCATCAGGTCTTCATCG**–**3`		

The expression of the target genes in both treated and untreated samples was analyzed and normalized against *rpoD* expression. The level of gene expression in untreated and treated samples was calculated relative to the untreated PAO1.

### 2.8 Molecular Docking

CFP, CFPC, and CFPF were docked into the active site of *P. aeruginosa* ligand-binding domain at PDB ID: 1RO5 (Gould et al., 2004), PDB ID: 2UV0 (Bottomley et al., 2007) and PDB ID: 4JVD (Ilangovan et al., 2013) to evaluate their binding affinities, and to determine their inhibition activities and binding modes at the active site of LasI, LasR, and PqsR, respectively. The crystal structures of LasI, LasR, and PqsR were picked up from the protein data bank. All bound water ligands were removed from the protein. All components were constructed on ChemBioDraw using ChemBioOffice ultra v.14 software “ChemOffice, scientific personal productivity tools – PerkinElmer Informatics”. The energy was minimized by using MM2, Jop Type. Docking studies were performed using the Molsoft program, internal coordinate mechanics (ICM) 3.4-8C was applied as reported by Abagyan et al. (1994) by converting PDB file 1RO5, 2UV0, and 4JVD into an ICM object. ICM aims to find the global minimum energy that describes the interaction between the ligand and the receptor. The modes of the interaction of the autoinducer molecule 3-oxo-C12-HSL within 2UV0 and 2-nonyl-4-hydroxyquinoline (NHQ) within 4JVD were used as standard docked models.

### 2.9 Statistical Analysis

Each experiment was performed in triplicate. Mean and standard deviations of three independent measurements were calculated by Excel data package. Statistical analysis was performed using the GraphPad Instate software package (version 3.1) using T paired test for comparing the treated and untreated cultures. The results were assigned as significant when *p ≤*0.05, and highly significant when *p ≤*0.01 or *p ≤*0.001.

## 3 Results

### 3.1 QSI of Metallic-Cefoperazone Derivatives Using *C. violaceum* ATCC 12472

CFP, CFPF, and CFPC reduced violacein pigment production as indicated in table (1). The diameters of inhibition of violacein pigment were 25, 33, and 34 mm for CFP, CFPF, and CFPC, respectively (**Table 1** **)** (**Supplementary Figure 1**). However, CFP derivatization with MnCl_2_, NiCl_2,_ and CrCl_3_ did not exhibit QSI activity (**Supplementary Figure 1**). Therefore, CFP and metallic-complexes CFPF, and CFPC were selected to study their effects on virulence factors of *P. aeruginosa* clinical isolates and standard strains.

### 3.2 Minimal Inhibitory Concentrations of CFP, CFPF, and CFPC

Minimal inhibitory concentrations of CFP, CFPF, and CFPC against *P. aeruginosa*; Ps1, Ps2, Ps3, PAO1, PA14, and PAO-JP2 were evaluated. All tested strains were resistant to CFP with MIC 512 μg/mL. CFPC showed a prominent antimicrobial activity with low MICs of 2, 4, 8, 8, 8, and 32 μg/mL against tested strains: Ps1, Ps2, Ps3, PAO1, PA14, and PAO-JP2, respectively. In addition, MICs of CFPF were 2, 32, 128, 16, 64, and 16 μg/mL against *P. aeruginosa* strains: Ps1, Ps2, Ps3, PAO1, PA14, and PAO-JP2, respectively (**Table 3**).

**Table 3:** MICs and sub-MICs; 1/2 and 1/4 of Cefoperazone (CFP), Cefoperazone-Cobalt complex (CFPC) and Cefoperazone-Iron complex (CFPF).

	CFP			CFPC			CFPF		
	MIC μg/mL	1/2 MIC μg/mL	1/4 MIC μg/mL	MIC μg/mL	1/2 MICμg/mL	1/4 MIC μg/mL	MIC μg/mL	1/2 MIC μg/mL	1/4 MIC μg/mL
***P. aeruginosa* Ps1**	512	256	128	2	1	0.5	2	1	0.5
***P. aeruginosa* Ps2**	512	256	128	4	2	1	32	16	8
***P. aeruginosa* Ps3**	512	256	128	8	4	2	128	64	32
***P. aeruginosa* PAO1**	512	256	128	8	4	2	16	8	4
***P. aeruginosa* PA14**	512	256	128	8	4	2	64	32	16
***P. aeruginosa* PAO-JP2**	512	256	128	32	16	8	16	8	4

### 3.3 Effect of Sub-Inhibitory Concentrations of the Tested Compounds on the Growth of *P. aeruginosa* Isolates

Viable count of *P. aeruginosa* was estimated after treating each isolate with 1/2 MIC of CFP, CFPC, and CFPF. Cultivation of *P. aeruginosa* with sub-MICs did not affect bacterial growth when compared to the control untreated cultures (**Supplementary Table S1**). For instance, the viable count of Ps1 isolate was 152, 155 and 160 × 10^7^ CFU/mL when treated with 1/2 MIC of CFP, CFPC, and CFPF, respectively and the bacterial count of untreated Ps1 was 162× 10^7^ CFU/mL. The counts of Ps2 treated with 1/2 MIC of CFP, CFPC, and CFPF were 140, 145 and 138 ×10^6^ CFU/mL, respectively while the count of untreated culture was 146× 10^6^ CFU/mL. Also, the viable count of Ps3 was 132, 129, and 134 ×10^7^ CFU/mL upon addition of 1/2 MIC of CFP, CFPC, and CFPF, respectively while the count of untreated culture was 136× 10^7^ CFU/mL.

Furthermore, the viable count of PAO1 treated with 1/2 MIC of CFP, CFPC, and CFPF were 166, 160, and 164 ×10^7^ CFU/mL, respectively and the viable count of untreated PAO1 was 168× 10^7^ CFU/mL. Similarly, PA14 count was 175, 168, and 166 ×10^7^ CFU/mL when cultivated in 1/2 MIC of CFP, CFPC, and CFPF, respectively and the viable count of the untreated culture was 177× 10^7^ CFU/mL. The viable count of PAO-JP2 culture was 181, 184, 180 ×10^7^ CFU/mL when cultivated with 1/2 MIC of CFP, CFPC, and CFPF, respectively and the viable count of the untreated culture was 187× 10^7^CFU/mL.

Additionally, the OD 600 nm of treated and untreated cultures was measured at different time intervals. There was no effect on the bacterial growth over time in cultures treated with 1/2 MIC of CFP, CFPC, and CFPF compared to untreated cultures (**Supplementary Figure S2**).

### 3.4 Effect of Sub-MICs of the Tested Compounds on Virulence Factors

The influences of sub-MICs; 1/2 and 1/4 of CFP, CFPC, and CFPF on the virulence factors; pyocyanin, protease, hemolysin, and biofilm of *P. aeruginosa* clinical isolates Ps1, Ps2 and Ps3, and standard strains PAO1, PA14, and PAO-JP2 were investigated and compared with untreated cultures at the same conditions.

#### 3.4.1 Pyocyanin Assay

**Figure 1:**
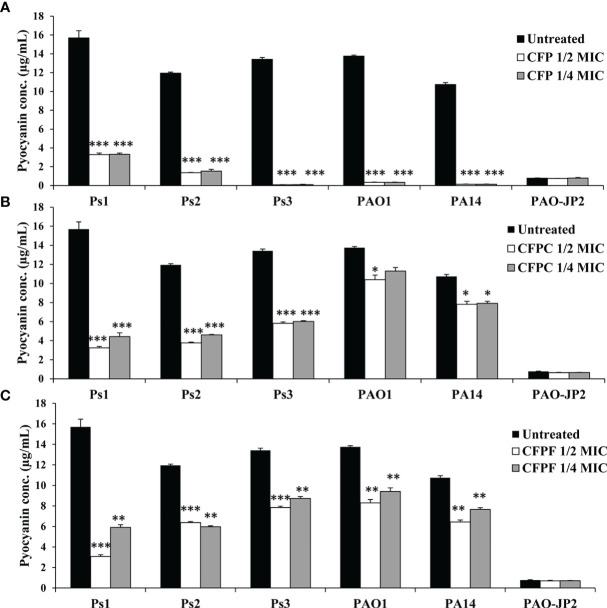
Pyocyanin production; Effect of sub-MICs; 1/4 and 1/2 of Cefoperazone (CFP) **(A)** Cefoperazone-Cobalt complex (CFPC) **(B)** and Cefoperazone-Iron complex (CFPF) **(C)** on pyocyanin production in *P. aeruginosa* strains, Ps1, Ps2, Ps3, PAO1, PA14 and PAO-JP2 (negative control) compared to the untreated cultures. Error bars represent S.D. (n = 3), *p ≤ 0.05, **p ≤ 0.01, and ***p ≤ 0.001.

CFP at 1/2 MIC (256 μg/mL) significantly (*p <*0.001) inhibited pyocyanin production in *P. aeruginosa* strains; Ps1, Ps2, Ps3, PAO1 and PA14 by 79.0%, 88.6%, 99.4%, 97.5% and 98.7%, respectively. Also, 1/4 MIC of CFP reduced pyocyanin by 78.9%, 87.2%, 99.3%, 97.5% and 98.7% in Ps1, Ps2, Ps3, PAO1 and PA14, respectively (*p <*0.001). Interestingly, CFPC at a significantly low concentrations (1-16 μg/mL) reduced pyocyanin production by 79.4%, 68.5%, 56.6%, 24.4% and 25.5% in Ps1, Ps2, Ps3, PAO1 and PA14, respectively. Moreover, 1/4 MIC (0.5, 1, 2, 2 and 2 μg/mL) of CFPC significantly lowered pyocyanin levels by 71.8%, 61.4%, 55.1%, and 25% in Ps1, Ps2, Ps3, and PA14, respectively. On the same instance, 1/2 MIC of CFPF significantly inhibited pyocyanin production by 80.4%, 46.7%, 45.2%, 41.6%, 39.7% and 40.1% in Ps1, Ps2, Ps3, PAO1 and PA14 cultures, respectively. Furthermore, 1/4 MIC of CFPF (0.5, 8, 32, 4 and 16 μg/mL) significantly reduced pyocyanin by 62.4%, 50.0%, 34.9%, 31.7% and 28.8% in Ps1, Ps2, Ps3, PAO1 and PA14, respectively (*p <*0.001 and *p <*0.01) (**Figure 1**).

#### 3.4.2 Total Protease Assay

**Figure 2:**
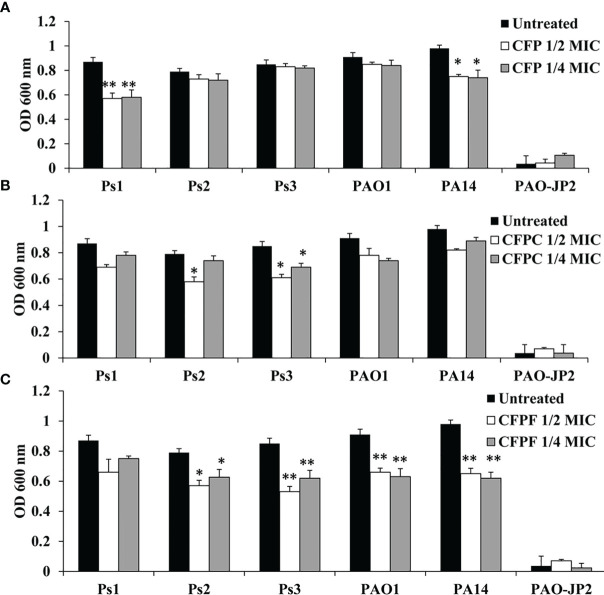
Protease production; Effect of sub-MICs; 1/4 and 1/2 of Cefoperazone (CFP) **(A)** Cefoperazone-Cobalt complex (CFPC) **(B)** and Cefoperazone-Iron complex (CFPF) **(C)** on total protease production in *P. aeruginosa* strains, Ps1, Ps2, Ps3, PAO1, PA14 and PAO-JP2 (negative control) compared to the untreated cultures. Error bars represent S.D. (n = 3), *p ≤ 0.05, and **p ≤ 0.01.

CFP at 1/2 MIC reduced protease production by 34.5% (*p <*0.01), 7.6%, 2.4%, 6.6% and 23.5% (*p <*0.05) in Ps1, Ps2, Ps3, PAO1 and PA14 strains, respectively. *Pseudomonas* isolates Ps1, Ps2, Ps3, PAO1 and PA14 supplied with 1/4 MIC exhibited decrease in protease levels by 33.3% (*p <*0.01), 8.9%, 3.5%, 7.7% and 24.5% (*p <*0.05), respectively. CFPC at 1/2 MIC inhibited proteolytic activity by 20.7%, 26.6%, 28.2%, 14.3% and 16.3% in Ps1, Ps2, Ps3, PAO1 and PA14, respectively. CFPC at 1/4 MIC reduced protease by 10.3%, 6.3%, 18.8%, 18.7% and 9.2% in Ps1, Ps2, Ps3, PAO1 and PA14, respectively. Sub-MICs of CFPF at 1/2 MIC lowered protease activity by 24.1%, 28%, 37.6%, 27.5% and 33.7% in Ps1, Ps2, Ps3, PAO1 and PA14, respectively. CFPF at 1/4 MIC reduced protease by 13.8%, 20.7%, 27.1%, 30.8% and 36.7% in Ps1, Ps2, Ps3, PAO1 and PA14, respectively. PAO-JP2 showed the least protease activity of 0.036 at OD 600 nm (**Figure 2**).

#### 3.4.3 Hemolysin Activity

**Figure 3:**
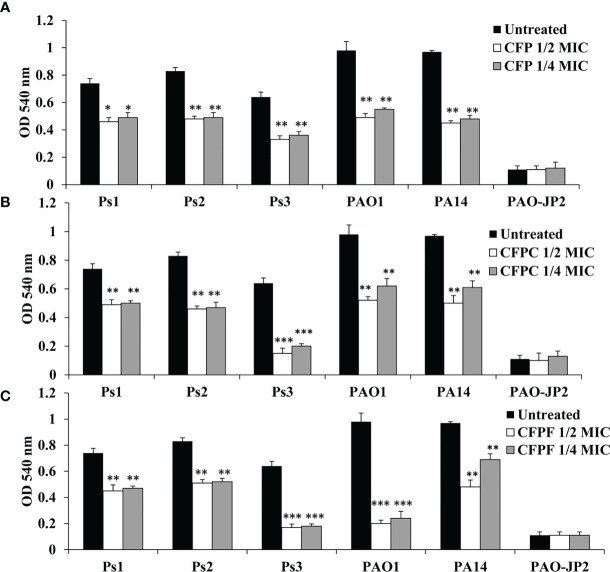
Hemolysin activity; Effect of sub-MICs; 1/4 and 1/2 of Cefoperazone (CFP) **(A)** Cefoperazone-Cobalt complex (CFPC) **(B)** and Cefoperazone-Iron complex (CFPF) **(C)** on hemolysin activity measured at OD 540 nm in *P. aeruginosa* strains, Ps1, Ps2, Ps3, PAO1, PA14 and PAO-JP2 (negative control) compared to the untreated cultures. Error bars represent S.D. (n = 3), *p ≤ 0.05, **p ≤ 0.01, and ***p ≤ 0.001.

Sub-MICs of CFP significantly inhibited hemolysin production in Ps1, Ps2, Ps3, PAO1 and PA14 by 38%, 42.2%, 48.4%, 50% and 53.6%, respectively when supplied with 1/2 MIC (256 μg/mL). Adding CFP at 1/4 MIC (128 μg/mL) significantly (*p* < 0.01) reduced hemolysin by 33.8%, 41%, 43.8%, 49.8% and 50.5% in Ps1, Ps2, Ps3, PAO1 and PA14, respectively. CFPC at 1/2 MIC showed significant reduction (*p* < 0.01) in hemolysin activity by 33.8%, 44.6%, 76.6%, 46.9% and 48.5% in Ps1, Ps2, Ps3, PAO1 and PA14, respectively. Also, 1/4 MIC significantly (*p <*0.01) inhibited hemolysin by 32.4%, 43.4%, 68.8%, 36.7% and 37.1% in Ps1, Ps2, Ps3, PAO1 and PA14, respectively. Similarly, 1/2 MIC of CFPF significantly reduced hemolysin by 56.9%, 60.4%, 73.4%, 79.6% and 50.5% in Ps1, Ps2, Ps3, PAO1 and PA14, respectively. Also, 1/4 MIC of CFPF reduced hemolysin by 39.2%, 38.6%, 73.4%, 79.6% and 50.5% in Ps1, Ps2, Ps3, PAO1 and PA14, respectively (*p <*0.01). The negative control PAO-JP2 exhibited the least hemolysin activity as 0.11 at OD 540 nm (**Figure 3**).

#### 3.4.4 Effect on Biofilm Formation

**Figure 4:**
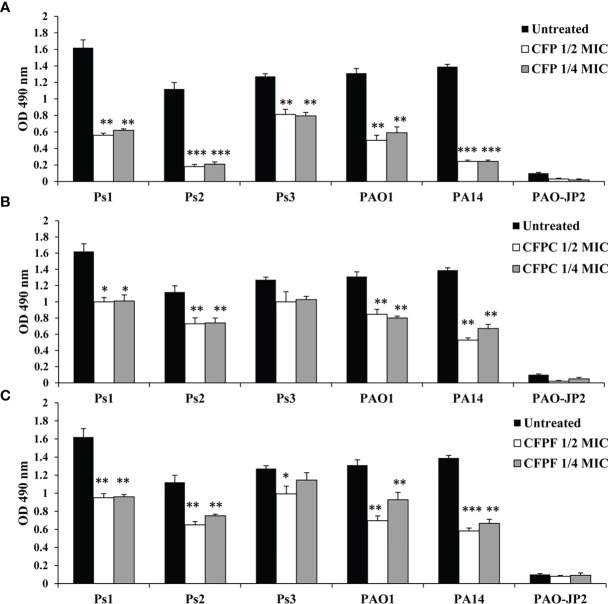
Biofilm formation; Effect of sub-MICs; 1/4 and 1/2 of Cefoperazone (CFP) **(A)**, Cefoperazone-Cobalt complex (CFPC) **(B)** and Cefoperazone-Iron complex (CFPF) **(C)** on biofilm formation in *P. aeruginosa* strains, Ps1, Ps2, Ps3, PAO1, PA14 and PAO-JP2 (negative control) compared to the untreated cultures. Error bars represent S.D. (n = 3), *p ≤ 0.05, **p ≤ 0.01, and ***p ≤ 0.001.

Using 1/2 MIC of CFP significantly (*P <*0.01) inhibited biofilm formation by Ps1, Ps2, Ps3, PAO1 and PA14 by 61.1%, 84%, 36.1%, 62% and 85.6%, respectively. Treatment with 1/4 MIC of CFP (128 μg/mL) eliminated biofilm by 56.9%, 81.3%, 37.6%, 54.8% and 82.5%, respectively. Surprisingly, CFPC at 1/2 MIC significantly reduced biofilm formation by 30.6%, 34.8%, 21.2%, 35.4% and 62% in Ps1, Ps2, Ps3, PAO1 and PA14, respectively. At the same instance, 1/4 MIC of CFPC significantly reduced biofilm by 29.9%, 34%, 19%, 38.9% and 51.8%, respectively. Furthermore, CFPF significantly diminished biofilm formation by Ps1, Ps2, Ps3, PAO1 and PA14 when supplied with 1/2 MIC by 34%, 42%, 22%, 46.9% and 58%, respectively. Using 1/4 MIC of CFPF reduced biofilm by 33.3%, 33%, 9.8%, 29.2% and 52.1% in *P. aeruginosa* strains; Ps1, Ps2, Ps3, PAO1 and PA14, respectively (**Figure 4**).

### 3.5 Expression of QS-Regulated Genes

**Figure 5:**
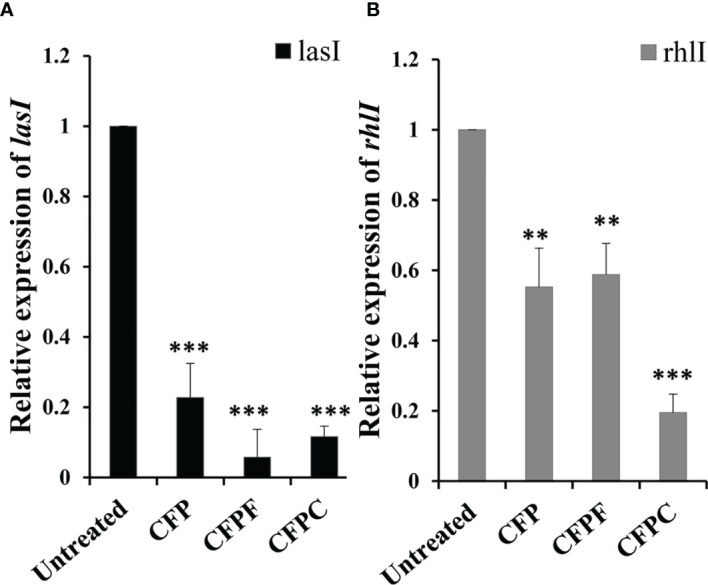
Cefoperazone (CFP), Cefoperazone-Cobalt complex (CFPC) and Cefoperazone-Iron complex (CFPF) at 1/2 MIC inhibited QS regulatory genes **(A)** *lasI* and **(B)** *rhlI* of *P. aeruginosa* PAO1 compared to the expression of genes in untreated PAO1. Error bars represent S.D. (n = 3) **p ≤ 0.01 and ***p ≤ 0.001.

To get insights into the molecular mechanism of CFP, CFPC and CFPF in reducing QS of *P. aeruginosa.* RT- PCR was used to assess relative expression levels of *lasI*, and *rhlI* genes. CFP, CFPC and CFPF was found to significantly (*p* < 0.001) reduce the transcription of *lasI* by 77.25%, 94% and 88%, respectively in PAO1 compared to the untreated samples (**Figure 5A**).

Also, significant inhibition in *rhlI* gene expression in *P. aeruginosa* PAO1 was elucidated when subjected to CFP, CFPF and CFPC at sub-MIC. The relative expression data showed a significant reduction in *rhlII* gene expression by 44% in CFP treated cells (*p* < 0.01), 41% reduction in CFPF treated culture (*p* < 0.01) and 80% reduction in CFPC treated cells (*p* < 0.001) when compared to the untreated cells (**Figure 5B**).

### 3.6 Binding Affinity Analysis for LasR and Ligands

**Figure 6:**
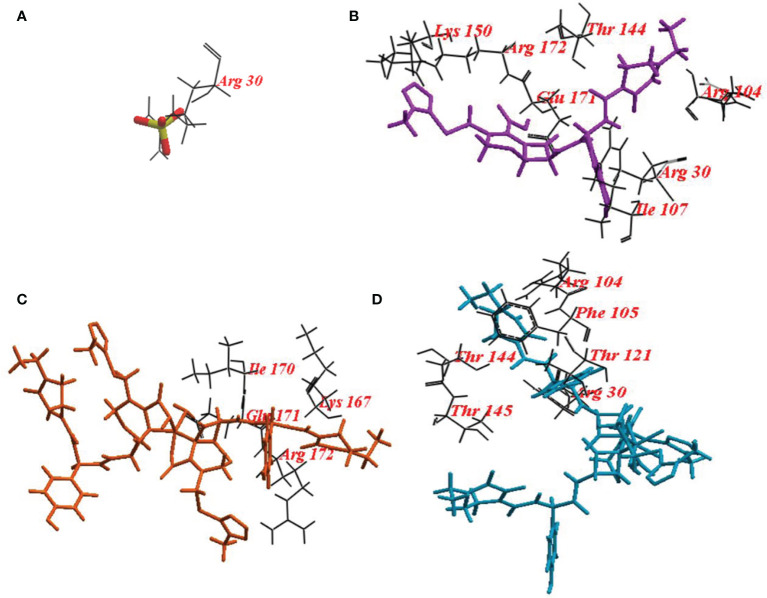
Binding mode of **(A)** redocked sulphate ligand; **(B)** Cefoperazone (CFP); **(C)**, Cefoperazone-Cobalt complex (CFPC) and **(D)** Cefoperazone-Iron complex (CFPF) into the Crystal structure *P. aeruginosa* acyl-homoserinelactone synthase LasI.

The ICM score and hydrogen bonds between compounds and the surrounding amino acids were used to predict the binding modes, their binding affinities, and the orientation of these compounds at the active site of LasI, LasR, and PqsR. The scoring functions of the compounds were calculated from minimized ligand-receptor complexes. CFP, CFPC and CFPF were docked at the binding site of LasI. CFP showed high ICM score of -123.78 and formed seventeen hydrogen bonds with Arg30, Arg104, Ile107, Thr144, Lys150, Arg172, and Glu171. Both derivatives; CFPC and CFPF gave higher ICM scores of -132.85 and -191.69, respectively (**Table 4**). CFPC binds with the receptor by ten hydrogen bonds with Lys167, Arg172, Ile170, and Glu171. While CFPF form nine hydrogen bonds with Arg30, Arg104, Thr144, Thr145, Phe105, and Thr121 (**Figure 6**, **Supplementary Table S2**).

**Table 4:** Internal coordinate mechanics scores, no. of H-bonds and amino acid residues involved in the interaction between LasR, LasI and PqsR binding site with tested compounds and the autoinducer molecule of the receptor.

Receptor	Compound	ICM Score	No. of H-Bonds	Amino acid residues involved
**LasI**	**Sulphate**	-44.03	12	Arg30.
	**CFP**	-123.78	17	Arg30, Arg104, Ile107, Thr144, Lys150, Arg172, Glu171.
	**CFPC**	-132.85	10	Lys167, Arg172, Ile170, Glu171.
	**CFPF**	-191.69	9	Arg30, Arg104, Thr144, Thr145, Phe105, Thr121.
**LasR**	**3-oxo-C12-HSL**	-107.47	3	Trp60, Asp73, Ser129.
	**CFP**	-112.96	10	Tyr47, Ala50, Arg61, Tyr64, Thr75, Gly126.
	**CFPC**	-136.44	26	Tyr47, Ala50, Gly54, Trp60, Arg61, Tyr64, Asp65, Asp73, Pro74, Thr75, Val76, Ser77, Ile86, Phe87, Gln98
	**CFPF**	-179.52	14	Tyr47, Asn49, Ala50, Gly54, Trp60, Arg61, Tyr64, Asp73, Thr75.
**PqsR**	**NHQ**	-57.78	3	Gln194, Ile236, Leu208.
	**CFP**	-105.37	14	Arg126, Ser128, Asp131, Thr135, Arg200, Asn220.
	**CFPC**	-130.05	17	Lys167, Asp264, Thr265, Lys266.
	**CFPF**	-161.85	28	Ser128, Ala130, Asp131, Ser132, Leu133, Ala134, Thr135, Gly198, Arg200, Ser201, Gln203, Ser205, Glu219.

**Figure 7:**
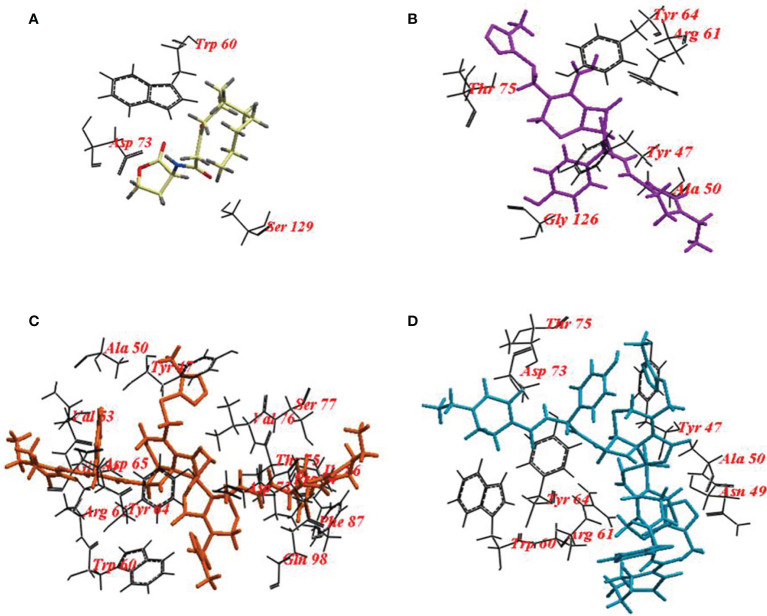
Binding mode of **(A)** 3-oxo-C12-HSL cefoperazone; **(B)** Cefoperazone (CFP); **(C)**, Cefoperazone-Cobalt complex (CFPC) and **(D)** Cefoperazone-Iron complex (CFPF) and redocked into the active site of *P. aeruginosa* ligand-binding domain.

To validate the docking protocol, the autoinducer 3-oxo-C12-HSL was docked in its binding site LasR, which revealed ICM score of -107.47 and form three hydrogen bonds with Trp60, Asp73, and Ser129. CFP showed ICM score of -112.96 and formed ten hydrogen bonds, one hydrogen bond with each of Tyr47, Tyr64, Thr75: two hydrogen bonds with each of Ala50 and Gly126 and three hydrogen bonds with Arg61. CFPC and CFPF gave ICM scores of -136.44 and -179.52, respectively which is higher than the autoinducer’s ICM score (-107.47) (**Table 4**). CFPC bound with the receptor by twenty-six hydrogen bonds with amino acids Tyr47, Ala50, Gly54, Trp60, Arg61, Tyr64, Asp65, Asp73, Pro74, Thr75, Val76, Ser77, Ile86, Phe87, and Gln98. While CFPF form fourteen hydrogen bonds with Tyr47, Asn49, Ala50, Gly54, Trp60, Arg61, Tyr64, Asp73, Thr75 (**Figure 7**, **Supplementary Table S3**).

**Figure 8:**
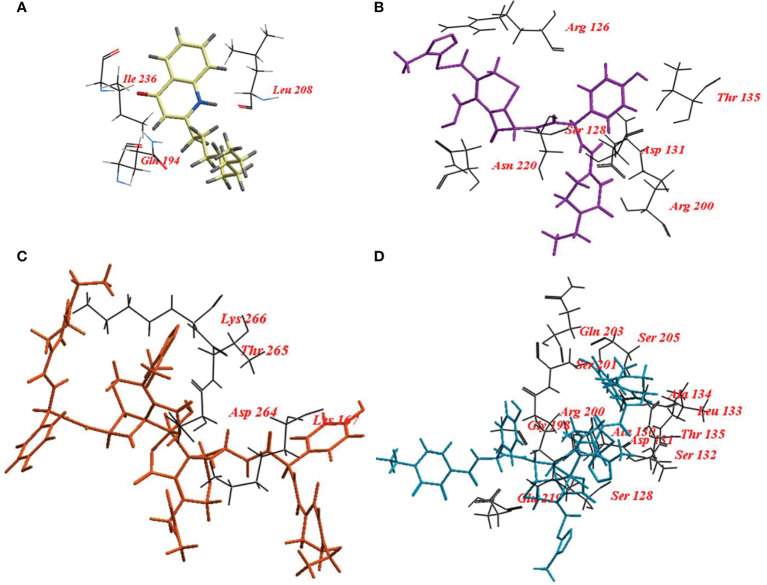
Binding mode of **(A)** 2-nonyl-4-hydroxyquinoline (NHQ) redocked ligand; **(B)** Cefoperazone (CFP); **(C)**, Cefoperazone-Cobalt complex (CFPC) and **(D)** Cefoperazone-Iron complex (CFPF) into the Crystal structure of PqsR coinducer binding domain of *P. aeruginosa*.

Also, NHQ was docked into its binding site PqsR, it revealed ICM score of -57.78 and formed three hydrogen bonds with Gln194, Ile236, and Leu208. CFP, CFPC and CFPF showed ICM score of -105.37, -130.05 and -161.85, respectively (**Table 4**). CFP formed fourteen hydrogen bonds with Arg126, Ser128, Asp131, Thr135, Arg200, and Asn220. CFPC bound to the receptor by seventeen hydrogen bonds with Lys167, Asp264, Thr265, and Lys266. While CFPF formed twenty-eight hydrogen bonds with Ser128, Ala130, Asp131, Ser132, Leu133, Ala134, Thr135, Gly198, Arg200, Ser201, Gln203, Ser205, and Glu219 (**Figure 8**, **Supplementary Table S4**).

Molecular docking of CFP, CFPC and CFPF with LasI, LasR, and PqsR of *P. aeruginosa* are indicated in the **Supplementary Tables S2–S4**.

## 4 Discussion

*P. aeruginosa* is a common opportunistic organism with a remarked ability for survival in various environmental conditions. QS is the key element that regulates gene expression and virulence behaviors such as proteases, hemolysin, pyocyanin pigment, and biofilm formation (Schuster and Greenberg, 2006; Thornton et al., 2020). Recently, treatment of *P. aeruginosa* infection represents a great challenge due to the high incidence of drug resistance. So, the development of a new therapeutic approach is imperative, one of these approaches is targeting QS cascade by inhibitors to control *Pseudomonas* virulence factors. Hence, bacterial infection can be easily eliminated by the immune system without the concern of microbial resistance (Hentzer and Givskov, 2003).

According to our knowledge, less is known about QSI activity of CFP, and no data is available about the antiquorum sensing effects of CFPC and CFPF. In this study, we evaluated the antiquorum sensing activities of CFP using *C. violaceum* ATCC 12472 reporter strain. *C. violaceum* ATCC 12472 produces several N-acyl-L-homoserine lactones (AHLs); *N*-nonanoyl-homoserine lactone, *N*-undecanoyl-homoserine lactone, *N*-(3-oxodecanoyl)-homoserine lactone (3-oxo-C10-HSL), N-(3-hydroxydecanoyl)-L-homoserine lactone, *N*-(3-hydroxyundecanoyl)-homoserine lactone, and 3-oxo-C12-HSL (Mion et al., 2021). N-(3-hydroxydecanoyl)-L-homoserine lactone is the main inducer that controls violacein production *via* quorum sensing. Other signaling molecules such as 3-oxo-C10-HSL and 3-oxo-C12-HSL can enhance the production of violacein in *C. violaceum* ATCC 12472 (Morohoshi et al., 2008). In this study, CFP, and its metallic derivatives CFPC and CFPF inhibited violacein pigment (**Table 1**) without any effect on microbial growth. Similarly, the QSI activity of flavonoid from *Cassia alata* (Rekha et al., 2017), quercetin and quercetin-3-O-arabinoside from *Psidium guajava* (Vasavi et al., 2014), curcumin, apigenin and luteolin (Burcu Bali et al., 2019), and enantiopure alkylglycerols (Chaverra Daza et al., 2021) inhibited violacein pigment production of *C. violaceum* ATCC 12472 and eliminated quorum sensing associated virulence factors of *P. aeruginosa.* QSI activity of antimicrobial agents has been previously reported including azithromycin (Bala et al., 2011), ciprofloxacin, tobramycin, and ceftazidime (Garske et al., 2004; Skindersoe et al., 2008).

Interestingly, CFPC and CFPF reduced QS-related virulence factors of *P. aeruginosa* without any effect on cell viability. For instance, *P. aeruginosa* produces green pyocyanin pigment after 24-48 hours of growth under the control of PQS and *rhlI/R* signaling systems (Gupta et al., 2011). CFP reduced pyocyanin levels in all *P. aeruginosa* strains both clinical isolates and standard strains after treatment by 1/2 and 1/4 MICs (128-256 µg/mL). At 2-8 folds low concentrations, CFPC (0.5-16 µg/mL) and CFPF (0.5-64 µg/mL) significantly reduced pyocyanin production to the level of PAO-JP2 (negative control) (**Figure 1**). Also, CFP, CFPC, and CFPF inhibited las-regulated virulence genes such as protease and hemolysin (**Figures 2**, **3**). These results are consistent with reports that β-lactams antibiotics such as cefepime, ceftazidime, and imipenem inhibit pyocyanin, protease, and hemolysin production in *P. aeruginosa* PAO1 without affection microbial viability (El-Mowafy et al., 2017; Aleanizy et al., 2021). Also, previous findings showed that metformin (Abbas et al., 2017) and zinc oxide nanoparticles (Saleh et al., 2019) inhibited hemolytic activity of *P. aeruginosa*.

Additionally*, P. aeruginosa* has a pronounced ability for biofilm assembly. *Pseudomonas* biofilms are resistant to most of the conventional antibiotic regimens. Inhibition of biofilm formation makes bacterium more susceptible to immune system and phagocytosis by neutrophils (Hentzer et al., 2002; Hentzer and Givskov, 2003). CFP, CFPC, and CFPF at 1/2 and 1/4 MICs showed a significant reduction in biofilm formation (**Figure 4**). Quorum sensing regulates the production of several extracellular virulence factors and promotes biofilm maturation, meaning that it has a key role in the pathogenesis of *P. aeruginosa* (Wagner et al., 2003). Azithromycin (Swatton et al., 2016), imipenem (El-Mowafy et al., 2017), piperacillin/tazobactam (Aleanizy et al., 2021) inhibit biofilm formation *via* interruption with the QS cascade. In addition, QSI activity of garlic renders *P. aeruginosa* biofilms sensitive to tobramycin and phagocytosis (Bjarnsholt et al., 2005).

On the molecular level, we tested the activity of CFP, CFPC, and CFPF on the expression of QS genes *lasI*, and *rhlI*. Data revealed that CFP, CFPC, and CFPF at 1/2 MIC exhibited significant reduction in the relative expression of *lasI* by 77.25%, 94% and 88%, respectively (**Figure 5**). The decrease in the expression level of *lasI* was consistent with reduction in all virulence factors controlled by *lasI/R*. The interaction of LasR with 3-oxo-C12-HSL, induces las system and triggers the production of virulence factors such as elastase, alkaline protease, hydrogen-cyanide synthase, exotoxin A, and secretion apparatus (Pessi et al., 2001). Previous researches elucidated that inhibition of *lasI/R* QS circuit is associated with reduction of *P. aeruginosa* virulence traits including elastase, total protease, hemolysin, and biofilm (Tateda et al., 2001; Gupta et al., 2011).

Also, the relative expression of *rhlI* was significantly reduced by CFP, CFPC, and CFPF (**Figure 5**). The *lasI/R* and *rhlI/rhlR* circuits are interconnected and suppression of *lasI* is associated with subsequent inhibition of *rhlI* gene expression (Pesci et al., 1997). The *rhl* system is also necessary for optimal production of lasB elastase, lasA protease, pyocyanin (Latifi et al., 1996). Furthermore, the *rhl* system is involved in the regulation of hcnABC, and alkaline protease (Pearson et al., 1997; Pessi et al., 2001).

For a deep understanding of the CFP, CFPC, and CFPF activities, docking of these compounds was performed at the active site of LasI, LasR, and PqsR. Docking study of LasI revealed higher ICM scores exhibited by CFP, CFPC and CFPF than that of the natural legend (**Table 4**). CFP bound the receptor with Arg30, Arg104, Ile107, Thr144, Lys150, Arg172, and Glu171. CFPC bind with Lys167, Arg172, Ile170, and Glu171. While CFPF bound with Arg30, Arg104, Thr144, Thr145, Phe105, and Thr121 (**Figure 6**). Several of these residues, including Met79, Phe105, Thr142 and Thr144, are well conserved among AHL-synthases. The basic residues, Lys150, Arg154, Arg161, His165, Lys167, and Arg172 form a highly positive charged patch on the LasI surface (Gould et al., 2004). Likewise, carvacrol interacts with LasI synthase Arg104 residue through Van der Waals forces and occupies the LasR binding site pocket at Asp73 residue, with subsequent elimination of the expression of virulence factors in *P. aeruginosa* (Tapia-Rodriguez et al., 2019).

The amino acid residues in the active site of LasR are Ala50, Tyr56, Trp60, Tyr64, Asp73, Thr75, Cys79, Gly126, Trp88, Tyr93, Ala105, Leu110, and Ser12 as indicated in the pdbsumb (PDB ID: 2UV0). The hydrogen bonds between the polar groups of the acyl-homoserine lactone and residues Tyr56, Trp60, Asp73, and Ser129 of LasR are pivotal for the correct folding of LuxR protein (Monterrosa et al., 2019; Prateeksha et al., 2019; Hnamte et al., 2020). Ahmed and coauthors elucidate the binding of 3-oxo-C12-HSL with LasR *via* hydrogen bonds with Tyr56, Trp60, and Asp73 (Ahmed et al., 2020). In our study, 10 hydrogen bonds were formed between CFP and LasR receptor at Tyr47, Tyr64, Thr75, Ala50, Gly126, and Arg61 residues. The binding energy of CFP (-122.96), CFPC (-141.38) and CFPF (-179.52) with LasR was higher than that of 3-oxo-C12-HSL (-107.47) (**Figure 5** and **Table 4**). Therefore, the high binding affinity of CFPC and CFPF with LasR receptors could eliminate the binding of natural ligand 3-oxo-C12-HSL with LasR receptors (**Figure 7**).

As LasR is one of the main players in the QS circuits, inhibition of LasR is associated with subsequent inhibition of virulence factors controlled by lasI/R (protease, hemolysin) and pqs/rhl (pyocyanin and biofilm) (Schuster and Greenberg, 2006). Likewise, ibuprofen binds with LasR by hydrogen bonds at Asp73, Thr75, Thr115, and Ser129 amino acids with significant elimination of pyocyanin, rhamnolipid, and elastase (Dai et al., 2019). Also, 6-Gingerol has formed hydrogen bonding with Trp60, Arg61, and Tyr93 by hydrophobic interactions with Leu40, Tyr47, Ala50, Ala70, Val76, and Ala127 with significant inhibition of QS and virulence factors (Kim et al., 2015).

Moreover, NHQ binds to its receptor PqsR by amino acids Pro238, Thr265, Ala237, Ala168, Phe221, Ile149, Ile236, Pro129, Ala130, Leu197, Leu207, and Leu208. Molecular docking of nanolipoidal α-terpineol showed that α-terpineol has modulated QS-regulated virulence and biofilm formation in *P. aeruginosa* through competitively binding to LasR, RhlR and Pqs, and QS receptors (Bose et al., 2020).

Hence, the tested compounds CFP, CFPC, and CFPF when docked into the active sites of LasI, LasR and PqsR receptors, they bound with the important amino acids with high ICM scores (**Figures 6**-**8**). This indicates that the tested compounds are potential inhibitors of QS and related virulence factors. Data is also supported by phenotypic and molecular studies.

In conclusion, this study explores a new character of CFP to comprise significant inhibition of QS and elimination of associated virulence factors. CFPC and CFPF at 2-8 folds lower concentration than CFP significantly eliminated QS and virulence factors of *P. aeruginosa* including (1) pyocyanin production (2) hemolysin (3) protease and (4) biofilm formation without affecting microbial growth. The interpretation of QSI activity of CFP, CFPC and CFPF was assessed by the molecular model analysis. It may be useful to investigate *in vivo* activity of CFPC and CFPF in the treatment of *P. aeruginosa* infections.

## Data Availability Statement

The original contributions presented in the study are included in the article/**Supplementary Material**. Further inquiries can be directed to the corresponding author.

## Author Contributions

All authors listed have made a substantial, direct, and intellectual contribution to the work, and approved it for publication.

## Conflict of Interest

The authors declare that the research was conducted in the absence of any commercial or financial relationships that could be construed as a potential conflict of interest.

## Publisher’s Note

All claims expressed in this article are solely those of the authors and do not necessarily represent those of their affiliated organizations, or those of the publisher, the editors and the reviewers. Any product that may be evaluated in this article, or claim that may be made by its manufacturer, is not guaranteed or endorsed by the publisher.
