# Supplementary material for: Quorum Sensing Inhibiting Activity of Cefoperazone and Its Metallic Derivatives on Pseudomonas aeruginosa
Source: Front Cell Infect Microbiol. 2021 Sep 30;11:716789. doi: 10.3389/fcimb.2021.716789 (PMC8515130; doi:10.3389/fcimb.2021.716789)
Supplement: Supplementary file 1 [file DataSheet_1.docx]

Supplementary Material

Quorum Sensing Inhibiting Activity of Cefoperazone and its Metallic Derivatives on *Pseudomonas aeruginosa*

**Nourhan G. Naga^1^, Dalia E. El-Badan^1^, Heba S. Rateb^2^, Khalid M. Ghanem^1^, Mona I. Shaaban^3*^**

^1^ Department of Botany and Microbiology, Faculty of Science, Alexandria University, Egypt

^2^ Department of Pharmaceutical and Medicinal Chemistry, Pharmacy College, Misr University for Science and Technology, Cairo, Egypt.

^3^ Department of Microbiology and Immunology, Faculty of Pharmacy, Mansoura University, Egypt

*** Correspondence:**

Mona Shaaban.

[mona_ibrahem@mans.edu.eg](mailto:mona_ibrahem@mans.edu.eg)

Table S1. Viable count of *P. aeruginosa* strains treated with 1/2 MIC of Cefoperazone (CFP), Cefoperazone-Cobalt complex (CFPC) and cefoperazone-Iron complex (CFPF).

|  | **Un-treated**  **CFU/mL** | **CFP**  **(CFU/mL)** | **CFPC**  **(CFU/mL)** | **CFPF**  **(CFU/mL)** |
| --- | --- | --- | --- | --- |
| ***P. aeruginosa* Ps1** | 162× 10^7^ | 152× 10^7^ | 155× 10^7^ | 160× 10^7^ |
| ***P. aeruginosa* Ps2** | 146×10^6^ | 140×10^6^ | 145×10^6^ | 138×10^6^ |
| ***P. aeruginosa* Ps3** | 136×10^7^ | 132×10^7^ | 129×10^7^ | 134×10^7^ |
| ***P. aeruginosa* PAO1** | 168 ×10^7^ | 166 ×10^7^ | 160×10^7^ | 164×10^7^ |
| ***P. aeruginosa* PA14** | 177× 10^7^ | 175 ×10^7^ | 168×10^7^ | 166×10^7^ |
| ***P. aeruginosa* PAO-JP2** | 187×10^7^ | 181 ×10^7^ | 184×10^7^ | 180×10^7^ |

**Table S2. Molecular docking results of cefoperazone (CFP), Cefoperazone-Cobalt complex (CFPC) and Cefoperazone-Iron complex (CFPF) with interacting amino acids with LasI of *P. aeruginosa***

| **Drug** | **ICM Score** | **H-Bond** | **Amino acid residues** | **Atom of amino acid** | **Atom of comp.** | **length Å** |
| --- | --- | --- | --- | --- | --- | --- |
| CFP | -123.78 | 17 | Arg30 | hh21 | o4 | 1.58 |
|  |  |  | Arg30 | hh21 | o7 | 1.18 |
|  |  |  | Arg30 | hh22 | o7 | 2.39 |
|  |  |  | Arg30 | hh22 | o7 | 2.29 |
|  |  |  | Arg104 | hh11 | o8 | 2.63 |
|  |  |  | Ile 107 | hn | o5 | 1.6 |
|  |  |  | Thr144 | hg1 | o5 | 1.47 |
|  |  |  | Lys 150 | hz1 | n5 | 2.68 |
|  |  |  | Lys 150 | hz2 | n4 | 2.55 |
|  |  |  | Lys 150 | hz2 | n5 | 1.6 |
|  |  |  | Arg172 | hn | o1 | 2.37 |
|  |  |  | Arg172 | hh21 | n3 | 2.79 |
|  |  |  | Arg172 | hh21 | n4 | 1.99 |
|  |  |  | Arg172 | hh22 | n3 | 2.01 |
|  |  |  | Arg172 | hh22 | n4 | 2.23 |
|  |  |  | Glu171 | o | h17 | 2.12 |
|  |  |  | Ile107 | oe2 | h2 | 1.19 |
| CFPC | -132.85 | 10 | Lys167 | hn | o16 | 2.5 |
|  |  |  | Arg172 | hn | o12 | 2.41 |
|  |  |  | Arg172 | hh21 | n14 | 2.17 |
|  |  |  | Arg172 | hh22 | n13 | 2.11 |
|  |  |  | Arg172 | hh22 | n14 | 1.71 |
|  |  |  | Ile170 | o | h34 | 1.38 |
|  |  |  | Glu171 | oe1 | h54 | 0.94 |
|  |  |  | Glu171 | oe1 | h55 | 0.86 |
|  |  |  | Glu171 | oe2 | h54 | 1.67 |
|  |  |  | Glu171 | o | h44 | 2.42 |
| CFPF | -191.69 | 9 | Arg30 | hh11 | o11 | 1.89 |
|  |  |  | Arg30 | hh12 | o4 | 2.71 |
|  |  |  | Arg30 | hh12 | o11 | 2.63 |
|  |  |  | Arg104 | hn | o8 | 2.71 |
|  |  |  | Arg104 | hh11 | o8 | 1.99 |
|  |  |  | Thr144 | hg1 | o6 | 1.56 |
|  |  |  | Thr145 | hg1 | o16 | 2.67 |
|  |  |  | Phe105 | o | h19 | 2.27 |
|  |  |  | Thr121 | og1 | h18 | 2.43 |

**Table S3. Molecular docking results of** **Cefoperazone (CFP), Cefoperazone-Cobalt complex (CFPC) and Cefoperazone-Iron complex (CFPF) with interacting amino acids with LasR of P. aeruginosa**

| **Drug** | **ICM Score** | **H-Bond** | **Amino acid residues** | **Atom of amino acid** | **Atom of comp.** | **length Å** |
| --- | --- | --- | --- | --- | --- | --- |
|  |  |  |  |  |  |  |
| CFP | -112.96 | 10 | Ala 50 | hn | o7 | 2.43 |
|  |  |  | Ala 50 | hn | o8 | 2.15 |
|  |  |  | Arg 61 | hh21 | o3 | 2.2 |
|  |  |  | Arg 61 | hh22 | o2 | 2.49 |
|  |  |  | Arg 61 | hh22 | o3 | 1.84 |
|  |  |  | Tyr 64 | hh | o1 | 1.79 |
|  |  |  | Thr 75 | hg1 | n3 | 2.3 |
|  |  |  | Gly126 | hn | o5 | 2.77 |
|  |  |  | Tyr 47 | o | h18 | 1.78 |
|  |  |  | Gly126 | o | h17 | 2.32 |
| CFPC | -136.44 | 26 | Ala50 | hn | o5 | 2.79 |
|  |  |  | Gly54 | hn | o8 | 2.45 |
|  |  |  | Trp60 | he1 | o9 | 2.07 |
|  |  |  | Arg61 | he | o4 | 2.64 |
|  |  |  | Arg61 | hh11 | o6 | 1.63 |
|  |  |  | Tyr64 | hh | o1 | 2.07 |
|  |  |  | Tyr64 | hh | o2 | 0.98 |
|  |  |  | Tyr64 | hh | o3 | 2.78 |
|  |  |  | Thr75 | hn | o12 | 1.15 |
|  |  |  | Thr75 | hg1 | o11 | 2.12 |
|  |  |  | Thr75 | hg1 | o12 | 2.62 |
|  |  |  | Val76 | hn | o12 | 1.93 |
|  |  |  | Ser77 | hg | o15 | 2.8 |
|  |  |  | Ser77 | hg | o16 | 2.75 |
|  |  |  | Ile86 | hn | o13 | 2.43 |
|  |  |  | Phe87 | hn | o13 | 2.52 |
|  |  |  | Gln98 | he22 | o15 | 2.54 |
|  |  |  | Gln98 | he22 | o16 | 1.81 |
|  |  |  | Tyr47 | o | h18 | 2.55 |
|  |  |  | Tyr64 | oh | h3 | 1.92 |
|  |  |  | Asp65 | od1 | h18 | 2.65 |
|  |  |  | Asp73 | od2 | h3 | 1.77 |
|  |  |  | Pro74 | o | h44 | 1.34 |
|  |  |  | Thr75 | og1 | h34 | 2.77 |
|  |  |  | Thr75 | o | h54 | 2.4 |
|  |  |  | Ile86 | og1 | h43 | 1.24 |
| CFPF | -179.52 | 14 | Asn 49 | hn | n4 | 1.97 |
|  |  |  | Ala 50 | hn | o10 | 2.47 |
|  |  |  | Trp 60 | he1 | o14 | 2.46 |
|  |  |  | Arg 61 | hh11 | o3 | 2.51 |
|  |  |  | Arg 61 | hh11 | o4 | 1.51 |
|  |  |  | Arg 61 | hh12 | o3 | 1.37 |
|  |  |  | Arg 61 | hh12 | o4 | 2.39 |
|  |  |  | Arg 61 | hh21 | o2 | 2.67 |
|  |  |  | Arg 61 | hh21 | o3 | 1.95 |
|  |  |  | Tyr 64 | hh | o12 | 1.92 |
|  |  |  | Thr 75 | hg1 | o15 | 2.56 |
|  |  |  | Tyr 47 | o | h3 | 2.74 |
|  |  |  | Gly 54 | o | h18 | 1.59 |
|  |  |  | Asp 73 | od2 | h44 | 2.16 |

**Table S4. Molecular docking results of Cefoperazone (CFP), Cefoperazone-Cobalt complex (CFPC) and Cefoperazone-Iron complex (CFPF) with interacting amino acids with PqsR of *P. aeruginosa***

| **Drug** | **ICM Score** | **H-Bond** | **Amino acid residues** | **Atom of amino acid** | **Atom of comp.** | **length Å** |
| --- | --- | --- | --- | --- | --- | --- |
| CFP | -105.37 | 14 | Arg126 | he | n3 | 2.78 |
|  |  |  | Arg126 | he | n4 | 1.78 |
|  |  |  | Arg126 | he | n5 | 2.77 |
|  |  |  | Arg126 | hh22 | n4 | 2.27 |
|  |  |  | Arg126 | hh22 | n5 | 1.92 |
|  |  |  | Ser128 | hn | o4 | 1.11 |
|  |  |  | Arg200 | hh11 | o6 | 1.74 |
|  |  |  | Arg200 | hh11 | o7 | 1.58 |
|  |  |  | Arg200 | hh12 | o6 | 1.37 |
|  |  |  | Arg200 | hh21 | o6 | 2.42 |
|  |  |  | Asn220 | hd21 | o3 | 2.72 |
| CFPC | -130.05 | 17 | Asp131 | od2 | h18 | 1.92 |
|  |  |  | Thr135 | og1 | h17 | 2.16 |
|  |  |  | Asn220 | od1 | h2 | 1.4 |
|  |  |  | Lys167 | hn | o12 | 2.34 |
|  |  |  | Lys167 | hz1 | n12 | 2.2 |
|  |  |  | Lys167 | hz1 | n13 | 1.99 |
|  |  |  | Lys167 | hz1 | n14 | 2.38 |
|  |  |  | Lys167 | hz1 | o11 | 2.18 |
|  |  |  | Lys167 | hz2 | o11 | 2.64 |
|  |  |  | Lys167 | hz3 | o2 | 2.66 |
|  |  |  | Lys167 | hz3 | o11 | 1.39 |
|  |  |  | Thr265 | hn | o1 | 2.01 |
|  |  |  | Lys266 | hz1 | o7 | 2.23 |
|  |  |  | Lys266 | hz1 | o8 | 1.85 |
|  |  |  | Lys266 | hz2 | o8 | 1.53 |
|  |  |  | Asp264 | od1 | h3 | 1.96 |
|  |  |  | Asp264 | od1 | h54 | 2.77 |
|  |  |  | Asp264 | od1 | h55 | 1.51 |
|  |  |  | Asp264 | od2 | h3 | 1.55 |
|  |  |  | Asp264 | od2 | h55 | 2.62 |
| CFPF | -161.85 | 28 | Ser128 | hn | o10 | 2.32 |
|  |  |  | Ser128 | hn | o9 | 0.85 |
|  |  |  | Ser128 | hn | n12 | 2.17 |
|  |  |  | Ser128 | hg | o11 | 1.82 |
|  |  |  | Ser132 | hn | o4 | 2.07 |
|  |  |  | Leu133 | hn | o4 | 2.08 |
|  |  |  | Ala134 | hn | o4 | 2.79 |
|  |  |  | Ala134 | hn | o6 | 1.52 |
|  |  |  | Thr135 | hn | o6 | 2.55 |
|  |  |  | Gly198 | hn | o12 | 1.99 |
|  |  |  | Arg200 | hn | o2 | 2.79 |
|  |  |  | Arg200 | hn | o3 | 1.85 |
|  |  |  | Arg200 | he | o1 | 1.2 |
|  |  |  | Arg200 | he | n3 | 2.05 |
|  |  |  | Arg200 | hh11 | o1 | 2.49 |
|  |  |  | Arg200 | hh22 | o1 | 2.52 |
|  |  |  | Arg200 | hh22 | n3 | 1.58 |
|  |  |  | Arg200 | hh22 | n4 | 1.73 |
|  |  |  | Ser201 | hg | o7 | 2.21 |
|  |  |  | Ser205 | hn | o5 | 1.83 |
|  |  |  | Glu219 | hn | o14 | 2.5 |
|  |  |  | Glu219 | hn | o15 | 2.34 |
|  |  |  | Ala130 | o | h9 | 1.86 |
|  |  |  | Asp131 | od1 | h9 | 2.54 |
|  |  |  | Asp131 | od2 | h3 | 2.35 |
|  |  |  | Gly198 | o | h34 | 0.95 |
|  |  |  | Arg200 | o | h43 | 1.37 |
|  |  |  | Gln203 | o | h18 | 2.06 |
|  |  |  | Arg126 | he | n3 | 2.78 |

**Supplementary figure S1.** Screening of quorum sensing inhibiting activity of Cefoperazone and its derivatives. using *Chromobacterium violaceum* ATCC 12427. Quorum sensing inhibiting activity of Cefoperazone and metallic derivatives (FeCl_3_.6H_2_O, CoCl_2_, MnCl_2_, NiCl_2_, and CrCl_3_was performed using *Chromobacterium violaceum* ATCC 12427.(A) CFP and its derivatives; (B) Cefoperazone-Iron complex (CFPF) and (C) Cefoperazone-Cobalt complex (CFPC) inhibited violet pigment of *Chromobacterium violaceum.* Cefoperazone derivatization with (D) MnCl_2_, (E) NiCl_2,_ and (F) CrCl_3_ did not exhibit QSI activity.

**Supplementary figure S2. Growth curve of *P. aeruginosa* strains** (A) Ps1, (B) Ps2, (C) Ps3, (D) PAO1, (E) PA14 and (F) PAO-JP2 in the presence and absence of 1/2 MIC of Cefoperazone (CFP), Cefoperazone-Iron complex (CFPF) and (C) Cefoperazone-Cobalt complex (CFPC).
